# Indole-3-Acetic Acid in *Burkholderia pyrrocinia* JK-SH007: Enzymatic Identification of the Indole-3-Acetamide Synthesis Pathway

**DOI:** 10.3389/fmicb.2019.02559

**Published:** 2019-11-05

**Authors:** Wan-Hui Liu, Fei-Fei Chen, Chao-En Wang, Huan-Huan Fu, Xue-Qi Fang, Jian-Ren Ye, Ji-Yuan Shi

**Affiliations:** Co-innovation Center for Sustainable Forestry in Southern China, College of Forestry, Nanjing Forestry University, Nanjing, China

**Keywords:** *Burkholderia pyrrocinia*, indole-3-acetamide, tryptophan 2-monooxygenase, indoleacetamide hydrolase, protein expression

## Abstract

*Burkholderia pyrrocinia* JK-SH007 is a plant growth-promoting bacteria (PGPB), that can promote the growth of poplar and other trees, and, production of the plant hormone indole-3-acetic acid (IAA) is one of the reasons for this effect. Therefore, the aims of this study were to evaluate the effect of the external environment on the synthesis of IAA by *B. pyrrocinia* JK-SH007 and to perform a functional analysis of its IAA synthesis pathway. In this study, IAA and its synthetic intermediates indole-3-acetamide (IAM), indole-3-pyruvic acid (IPyA), tryptamine (TAM), and indole-3-acetonitrile (IAN) were detected in *B. pyrrocinia* JK-SH007 fermentation broth by high-performance liquid chromatography and tandem mass spectrometry (HPLC-MS/MS), and these indolic compounds were also found in the cell-free extraction of *B. pyrrocinia* JK-SH007, but the genomic analysis of *B. pyrrocinia* JK-SH007 indicated that IAA biosynthesis was mainly through the IAM and TAM pathways. The effects of L-tryptophan (L-Trp), temperature and pH on the synthesis of IAA were investigated, and the results showed that L-Trp exerted a significant effect on IAA synthesis and that 37°C and pH 7 were the optimal conditions IAA production by *B. pyrrocinia* JK-SH007. In addition, the protein expression of tryptophan 2-monooxygenase and indoleacetamide hydrolase, which are the key enzymes of the indole acetamide-mediated IAA synthesis pathway, was analyzed, and their activity was verified by substrate feeding experiments. The results revealed the existence of an IAA synthesis pathway mediated by IAM and indicated that this pathway plays a role in *B. pyrrocinia* JK-SH007. This study lays the foundation for further exploration of the specific pathway and mechanism of IAA synthesis in *B. pyrrocinia* JK-SH007.

## Introduction

Plant growth-promoting bacteria (PGPB), which are ubiquitous in plants (Strobel et al., 2004), can help host plants absorb nutrients and improve their plant growth (Strobel et al., 2004; Santoyo et al., 2016). PGPB exert positive effects on plants via many mechanisms, including the production of indole-3-acetic acid (IAA), cytokinin, gibberellin, and ACC deaminase, nitrogen fixation, phosphorous solubilization and siderophore chelation of iron in soil (Glick, 2015; Olanrewaju et al., 2017; Ramakrishna et al., 2019). IAA is an auxin that plays an important role in plant growth and development (Benjamins and Scheres, 2008; Schaller et al., 2015) by stimulating cell elongation and rooting, increasing seed germination and seedling growth, and promoting plant growth at the shoot apex (Ahmad et al., 2005; Tsavkelova et al., 2007; Khamna et al., 2010; Zhang et al., 2017a, b). The ability to synthesize IAA is a common feature of PGPB (Olanrewaju et al., 2017), and although a large number of IAA-producing strains have been identified, studies on IAA biosynthesis-related genes or enzymes have generally been limited to a small number of model microorganisms, such as *Azospirillum* (Mandira and Srivastava, 2007), *Pseudomonas* (Patten and Glick, 2002a), and *Rhizobium* (Theunis et al., 2004). Various IAA biosynthetic pathways, including a tryptophan-dependent or tryptophan-independent pathways, have been proposed in plant-associated bacteria (Mcclerklin et al., 2018). The tryptophan-dependent pathways include the indole-3-acetamide (IAM), indole-3-pyruvic acid (IPyA), indole-3-acetonitrile (IAN), tryptamine (TAM), and tryptophan side-chain oxidase (TSO) pathways (Duca et al., 2014; Di et al., 2016), and the IAM pathway is one of the IAA synthesis pathways in which L-tryptophan (L-Trp) is a precursor. In this pathway, L-Trp is first converted into IAM by tryptophan 2-monooxygenase, which is encoded by the *iaaM* gene, and IAM is further catalyzed by indoleacetamide hydrolase, which is encoded by the *iaaH* gene, to synthesize IAA (Di et al., 2016). The IAM pathway is thought to exist in both phytopathogenic and beneficial bacteria (Gudrun et al., 1984; Theunis et al., 2004).

Indole-3-acetic acid production by bacteria is influenced by the culture conditions, growth stages and substrate availability, and the pressures exerted by different environmental conditions encountered by bacteria fine-tune the biosynthesis of this metabolite (Spaepen et al., 2007). The tryptophan concentration, pH, temperature, and carbon and nitrogen sources, among other variables, constitute the environmental conditions that are often encountered by bacteria, and these abiotic factors can change IAA biosynthesis (Ona et al., 2003, 2005; Malhotra and Srivastava, 2009; Molina et al., 2018). Therefore, the control of IAA biosynthesis requires a better understanding of mechanisms for optimizing these factors.

*Burkholderia pyrrocinia* JK-SH007 is an endophytic bacterium that was isolated from a branch of *Populus* × *euramericana* “Nanlin895” in Jiangsu Province, China, and belongs to the *Burkholderia cepacia* complex (Bcc) (Ren et al., 2011). This strain can significantly increase the enzymatic activity of poplar rhizosphere soil, which is conducive to the absorption of nutrients by plants and plays an important role in promoting poplar growth (Ren et al., 2011). In addition, this strain can colonize poplar, has endogenous characteristics, and can effectively inhibit the three pathogens causing poplar canker disease (*Cytospora chrysosperma*, *Phomopsis macrospora*, and *Fusicoccum aesculi*) (Ren et al., 2010, 2011, 2106). By increasing the ground diameter of pear trees, *B. pyrrocinia* JK-SH007 also promotes the growth of pear trees, improves the physiological indexes of leaves, increases the available phosphorus content in rhizosphere soil and improves fruit quality (Dou et al., 2017). Some species of Bcc have been widely reported as human opportunistic pathogens and are considered to be important pathogenic bacteria causing cystic fibrosis (CF) (Agodi et al., 2001; Campana et al., 2005; Mahenthiralingam et al., 2008). However, many Bcc strains are common biocontrol bacteria with many functions such as biological control, biodegradation, and plant growth (Bevivino et al., 2000; Sopheareth et al., 2013). Previous studies have shown that *B. pyrrocinia* JK-SH007 strain is not toxic to plant, animal or human (Ren, unpublished data), and it is in line with the safe application of Bcc. The strain does not contain the virulence genes *BCESM* and *cblA* (Ren et al., 2011), which is preliminarily proved to be biosafe. As a result, *B. pyrrocinia* JK-SH007 can be considered a potential PGPB (Ren et al., 2010, 2011, 2106).

However, the growth-promoting mechanism of *B. pyrrocinia* JK-SH007 remains unclear, although it is know that the synthesis of IAA plays a role in the growth-promoting effect of PGPB. A study of the characteristics and synthesis mechanism of the IAA produced by this strain can serve as a theoretical basis for understanding the growth-promoting effect of the strain on plants and thereby enhance its application in forests (Fan et al., 2018). Previous studies have shown that *B. pyrrocinia* JK-SH007 has the ability to synthesize IAA and that L-Trp exerts a significant effect on IAA synthesis. *B. pyrrocinia* JK-SH007 might harbor the IAM-mediated IAA synthesis pathway (Liu et al., 2019), but the conditions under which *B. pyrrocinia* JK-SH007 can synthesize IAA and the enzymes related to this pathway have not been elucidated. In this study, the ability of *B. pyrrocinia* JK-SH007 to synthesize IAA and the effects of different factors on its synthesis were analyzed, and the key rate-limiting enzymes in the IAA synthesis pathway of *B. pyrrocinia* JK-SH007 based on IAM, tryptophan 2-monooxygenase (*iaaM*) and indoleacetamide hydrolase (*iaaH*) were expressed and identified.

## Materials and Methods

### Experimental Materials

*Burkholderia pyrrocinia* JK-SH007 (strain number: CCTCC M209028), *Escherichia coli* BL21 (DE3) and the expression vector pET32a were provided by Jiangsu Key Laboratory for Prevention and Management of Invasive Species.

### Detection of IAA and Auxin Metabolites by HPLC-MS/MS

The *Burkholderia pyrrocinia* JK-SH007 strain was inoculated into tryptic soy broth (TSB) liquid medium at 1% and cultured with shaking at 30°C for 36 h to reach an OD_600__*nm*_ = 1.6). The fermentation broth was collected (filter with a 0.22 μm PES membrane filter unit) and sent to Nanjing Webiolotech Biotechnology Co., Ltd., for high-performance liquid chromatography and tandem mass spectrometry (HPLC-MS/MS) analysis.

### IAA Production From Cell-Free Extract of *B. pyrrocinia* JK-SH007

*Burkholderia pyrrocinia* JK-SH007 cells cultured for 36 h (OD_600__*nm*_ = 1.6) were collected, and the bacteria washed three times with potassium phosphate buffer (pH 7.5) and suspended. The mixed bacterial solution was treated with an ultrasonic cell crushing apparatus under an ice bath for 15 min. The bacterial body was then destroyed, and the contents were released (Shokri and Emtiazi, 2010). The supernatant containing acellular extract was obtained by centrifugation at 10 000 × *g* for 10 min. The supernatant was combined with solution of 1.0 mg/mL L-Trp (Yuanye, Shanghai, China). After 30 min of incubation at 37°C, the reaction mixtures were sent to Nanjing Webiolotech Biotechnology Co., Ltd., for HPLC-MS/MS analysis.

### Genomic Analysis of IAA Biosynthesis Pathways in *B. pyrrocinia* JK-SH007

The predicted and annotated gene sequences obtained from *B. pyrrocinia* JK-SH007 genome were analyzed by similarity analysis with KEGG (Kyoto Encyclopedia of Genes and Genomes) (Kanehisa et al., 2012) and NCBI (National Center for Biotechnology Information), and then each gene was allocated to the KEGG pathway maps of Trp metabolism and IAA biosynthesis. Based on this analysis, the whole IAA biochemical pathways was proposed and compared with the metabolite spectrum obtained by *B. pyrrocinia* JK-SH007 *in vitro* (Torres et al., 2018).

### Relationship Between Different Growth Periods of *B. pyrrocinia* JK-SH007 and IAA Biosynthesis

The *Burkholderia pyrrocinia* JK-SH007 strain was inoculated in TSB liquid medium at 1% and cultured at 30°C in the dark. The Microorganism IAA ELISA Kit (MLBIO, Shanghai, China) was used to determine the IAA content at 12, 16, 20, 24, 28, 32, 36, 40, 44, 48, 60, 72, 84, 96, 108, 120, 144, and 168 h, and the absorbances at OD_600__nm_ at these time points were determined.

### Effect of the L-Trp Concentration on IAA Production

An aseptic L-Trp solution (Yuanye, Shanghai, China) solution (obtained after removing bacteria using a 0.22 μm PES membrane filter unit) was added to TSB liquid medium and synthetic dropout (SD) liquid medium (18.22% D-Sorbitol, 0.67% yeast nitrogen base without amino acids, and 2% glucose, pH 5.8.) to final concentrations of 0.2, 0.5, 0.8, 1.0, 1.5, 2.0, 3.0, 4.0, 6.0, 8.0, and 10.0 mg/mL. TSB liquid medium or SD liquid medium without L-Trp solution was used as the control. *B. pyrrocinia* JK-SH007 was inoculated in the above mentioned media at 1%, and the IAA contents afterculture in these media for 48 h in the dark at 37°C were measured.

### Effects of Temperature and pH on the Synthesis of IAA by *B. pyrrocinia* JK-SH007

The aseptic L-Trp solution was added to TSB liquid culture medium to a final concentration of 0.5 mg/mL. The *B. pyrrocinia* JK-SH007 strain was inoculated into the culture medium at 1%, and the IAA contents and absorbances at OD_600__nm_ after culture with shaking at 15, 20, 25, 30, 37, and 42°C for 36 h in the dark were measured, 20 μL of *B. pyrrocinia* JK-SH007 was coated on TSB agar plates (containing 0.5 mg/mL L-Trp) and cultured at 15, 20, 25, 30, 37, and 42°C for 36 h as a control.

The pH value of TSB liquid culture medium was adjusted to 4, 5, 6, 7 and 8, and the aseptic L-Trp solution was then added to these culture media to a final concentration of 0.5 mg/mL. *B. pyrrocinia* JK-SH007 was inoculated into the culture media at 1%, and the IAA contents and OD_600__nm_ values after culture in these media for 36 h in the dark at 37°C were determined. Simultaneously, cultures of 20 μL of *B. pyrrocinia* JK-SH007 coated on TSB agar plates (containing 0.5 mg/mL L-Trp) with pH values of 4, 5, 6, 7, and 8, at 37°C for 36 h were used as a control.

### Prokaryotic Expression of Tryptophan 2-Monooxygenase and Indoleacetamide Hydrolase

#### Construction of the Prokaryotic Expression Vector

Based on the whole genome sequencing analysis and annotation results of *B. pyrrocinia* JK-SH007 (unpublished data), and according to the sequence characteristics of the *iaaM* and *iaaH* genes and the recognition site of the restriction endonucleases of the expression vector pET32a, *Eco*RV, and *Eco*RI restriction sites (underlined portions) were added to the forward and reverse primers for the target gene (designed with Primer 5.0). The primer sequences were as follows:

*iaaM*, E-iaaM-F: 5′-CGATATCATGCCGACGCACCGCTC GGCA-3′ and E-iaaM-R: 5′-CGAATTCTCAGTCGCCG TACCGATAGCG-3′; and *iaaH*, E-iaaH-F: 5′-CGA TATCATGCGCGAGATAGCCGATGC-3′ and E-iaaH -R: 5′-CGAATTCTCAGGCGGCCGTCGCGTGCT-3′.

The primers were synthesized by Nanjing GenScript Biotechnology Co., Ltd. The *iaaM* and *iaaH* genes were amplified using the *B. pyrrocinia* JK-SH007 genome DNA as the template and the high-fidelity enzyme PrimeSTARHS^®^ DNA Polymerase with GC Buffer (Takara, Dalian, China). The PCR system (50 μL) consisted of 25 μL of 2 × PrimeSTAR GC Buffer, 4 μL of dNTP mixture, 5 μL of Primer F/R, 2.5 μL of the DNA template, 0.5 μL of PrimeSTAR^®^ HS DNA Polymerase and 8 μL of ddH_2_O. PCR amplification was performed using the following conditions: 10 min at 95°C, 40 cycles of 30 s at 95°C, 30 s at 64°C (*iaaM*)/68°C (*iaaH*) and 2 min at 72°C, and 72°C for 10 min. The correctness of the PCR product was observed by 1% agarose gel electrophoresis, and the PCR product was purified by gel-excision using the TaKaRa MiniBEST Agarose Gel DNA Extraction Kit Ver. 4.0 (Takara, Dalian, China).

The purified products of the *iaaM* and *iaaH* genes and the expression vector pET32a were double-digested with the QuickCut restriction enzymes *Eco*RV and *Eco*RI (Takara, Dalian, China) and incubated at 37°C for 2 h. After reaction at 37°C for 2 h, the products were recovered by 1% agarose gel electrophoresis. The digested product of pET32a was ligated with the *iaaM* and *iaaH* gene digestion products overnight at 16°C using T4 DNA Ligase (Takara, Dalian, China). The recombinant expression plasmid was transformed into *E. coli* BL21 (DE3), plated on Luria-Bertani (LB) solid medium containing ampicillin (Amp) antibiotic (100 μg/mL), and grown overnight at 37°C. A positive recombinant was selected for colony PCR verification and double-enzyme digestion verification, and the positive recombinant was then sent to Beijing Genomics Institute (BGI) for sequencing.

#### Induced Expression and SDS-PAGE Analysis of Recombinant Proteins

*Escherichia coli* BL21 (DE3) with the recombinant plasmid pET32a-*iaaM/iaaH* and *E. coli* BL21 (DE3) with the empty vector pET32a (used as a control) was inoculated into LB liquid medium (containing 100 μg/mL Amp) at 1%. Once the OD_600__nm_ value reached 0.6, Isopropyl β-D-1-thiogalactopyranoside (IPTG) was added to a final concentration of 0.5 mM, and the culture was then induced overnight at 25°C. After centrifugation at 10 000 × *g* for 10 min, the supernatant was discarded, and the bacteria were washed twice with phosphate-buffered saline (PBS) buffer (10 mM, pH 7.2–7.4). The bacterial pellet was then resuspended, and for ultrasonic treatment, the resuspended bacterial liquid was crushed in an ice bath with a cell crushing apparatus until the optical density no longer increased and the bacterial suspension became clear. The supernatant and precipitate were collected for SDS-PAGE analysis.

Gene expression was induced overnight with 0.3 and 0.5 mM IPTG at 25°C under shaking conditions. The supernatant and precipitate after ultrasonic crushing were collected for SDS-PAGE analysis.

With an IPTG concentration of 0.5 mM, gene expression was induced after overnight incubation at temperatures of 15, 20, and 25°C. The supernatant and precipitate after ultrasonic crushing were collected for SDS-PAGE analysis.

#### Western Blot Analysis

The recombinant protein was subjected to SDS-PAGE electrophoresis, transferred to a PVDF membrane (0.45 μm), washed three times with PBST, blocked with a PBST solution containing 5% skimmed milk powder for 2 h, and incubated with 1:1 000 anti-His antibody (Tiangen Biotech, Beijing, China). The membrane was washed three times with PBST and added to anti-mouse IgG (H&L) AP conjugated (Promega, Madison, WI, United States) secondary antibody at a 1: 4 000 dilution. The membrane was incubated for 2 h at room temperature with gentle shaking and then washed three times with PBST, and color development was then induced by addition of the color development solution Western Blue^®^ Stabilized Substrate for Alkaline phosphatase (Promega, Madison, WI, United States). The reaction was terminated by rinsing the membrane with distilled water immediately after the color development met the requirements.

#### Purification and Renaturation of Tryptophan 2-Monooxygenase and Indoleacetamide Hydrolase

The supernatant of the tryptophan 2-monooxygenase fusion protein and the inclusion bodies of the indoleacetamide hydrolase fusion protein were purified using a Ni-IDA-Sefinose column (Sang Biotech, Shanghai, China). The purified indoleacetamide hydrolase fusion protein inclusion bodies were placed in dialysis bag (8–14 kD), and then placed in renaturation buffer [0.3 mol/L NaCl, 100 mmol/L NaH_2_PO_4_, 2 mmol/L reduced glutathione, 0.2 mmol/L oxidized L- glutathione, 40 mmol/L glycine, and 10% glycerol (v/v), pH 8.0], with decreasing concentrations of urea (6, 4, 2, and 1 mol/L) for 8 h. Finally, the solution was dialyzed in PBS buffer (10 mM, pH 7.2–7.4) without urea for 12 h. The solution in the dialysis bag was removed and centrifuged at 10 000 × *g* for 30 min (Xia et al., 2013). The purified and renatured proteins were concentrated by Amicon^®^ Ultra-15 centrifugal filtration (30 000 NMWL) and analyzed by SDS-PAGE. The protein concentration was determined with a BCA Protein Assay Kit (ComWin, Beijing, China).

#### Characterization of the Tryptophan 2-Monooxygenase and Indoleacetamide Hydrolase Enzymes

One assay mixture (1 mL) consisted of 1 mg of L-Trp, PBS buffer (10 mM, pH 7.2–7.4), and 0.2 mg of tryptophan 2-monooxygenase and incubated at 37°C for 2 h, and the other assay mixture (1 mL) consisted of 1 mg of IAM (Yuanye, Shanghai, China), PBS buffer (10 mM, pH 7.2–7.4), and 0.2 mg of indoleacetamide hydrolase and incubated at 37°C for 2 h. A mixture of the two above mentioned reactions was sent to Nanjing Webiolotech Biotechnology Co., Ltd., for HPLC-MS/MS analysis.

#### Statistical Analysis of the Data

The experimental data were analyzed by ANOVA, and Tukey’s post test using a significance level of *P* < 0.05. The values shown represent the standard deviations (SD) of the averages. All statistical analyses were performed using IBM SPSS Statistics 25.0 (IBM Inc., Armonk, NY, United States). Graphs were drawn using GraphPad Prism 6.0 (GraphPad Software, Inc., United States), and three biological replicates were included in each experiment.

## Results

### Detection of IAA and Auxin Metabolites by HPLC-MS/MS

Indole-3-acetic acid (Supplementary Figures S1A,C) and its synthetic intermediate, IAM (Supplementary Figures S1B,D), were detected in *B. pyrrocinia* JK-SH007 fermentation broth by HPLC-MS/MS analysis (a normal mass-to-charge ratio is obtained if the peak time of the sample is almost the same as or within a certain error from the peak time of the standard product, the mass-to-charge is normal), were detected in *B. pyrrocinia* JK-SH007 fermentation broth by HPLC-MS/MS analysis (Supplementary Figures S1C,D), which indicates that *B. pyrrocinia* JK-SH007 has the ability to synthesize IAA and that might harbor an IAM-mediated IAA synthesis pathway. In addition, the IAA precursors IPyA (Supplementary Figures S2A,B) IAN (Supplementary Figures S2C,D), and trace amounts of TAM (Supplementary Figures S2E,F) were detected (Table 1).

**TABLE 1:** HPLC-MS/MS analysis of indolic compounds produced by *B. pyrrocinia* JK-SH007 in TSB medium.

**Indolic compounds**		**Indole-3-acetic acid (IAA)**	**Indole-3-acetamide (IAM)**	**Indole-3-pyruvic acid (IPyA)**	**Indole-3-acetonitrile (IAN)**	**Tryptamine (TAM)**
Mass-to-charge ratio (M/Z)		176.0 ≥130.0	175.0 ≥130.0	202.0 ≥202.0	157.0 ≥117.0	161.0≥144.0
Acqusion	Standard	5.187	4.059	6.855	7.228	3.500
time (min)	Sample	5.187	4.063	6.915	7.050	3.589
Concentration (ng/mL)^a^		3.018 ± 0.270	0.782 ± 0.424	0.434 ± 0.293	0.431 ± 0.238	0.171 ± 0.058

*^*a*^Values are average ± *SD* (*n* = 3).*

### IAA Production by Cell-Free Extract

The cell-free extract of *B. pyrrocinia* JK-SH007 was incubated with L-Trp. IAA (Supplementary Figures S3A,C), IAM (Supplementary Figures S3B,D), IPyA (Supplementary Figures S4A,B), IAN (Supplementary Figures S4C,D) and TAM (Supplementary Figures S4E,F), in the reaction mixture were detected by HPLC-MS/MS (Table 2). These results showed the existence of enzymes related to IAA synthesis in *B. pyrrocinia* JK-SH007, that convert L-Trp into indole derivatives such as IAM and IAA. The existence of a tryptophan-dependent IAA synthesis pathway in *B. pyrrocinia* JK-SH007 was thus confirmed.

**TABLE 2:** HPLC-MS/MS analysis of indolic compounds produced in the cell-free extract of *B. pyrrocinia* JK-SH007.

**Indolic compounds**		**Indole-3-acetic acid (IAA)**	**Indole-3-acetamide (IAM)**	**Indole-3-pyruvic acid (IPyA)**	**Indole-3-acetonitrile (IAN)**	**Tryptamine (TAM)**
Mass-to-charge ratio (M/Z)		176.0 ≥130.0	175.0 ≥130.0	202.0 ≥202.0	157.0 ≥117.0	161.0 ≥144.0
Acqusion	Standard	6.868	5.856	7.614	7.051	4.517
time (min)	Sample	6.819	5.644	7.631	7.019	4.746
Concentration (ng/mL)^a^		1.044 ± 0.100	0.077 ± 0.040	0.526 ± 0.412	0.034 ± 0.010	0.275 ± 0.053

*^*a*^Values are average ± *SD* (*n* = 3).*

### Genomic Analysis of IAA Biosynthesis Pathways in *B. pyrrocinia* JK-SH007

The putative IAA biosynthetic pathways in the *B. pyrrocinia* JK-SH007 genome were identified based on the KEGG of Ttp metabolism and IAA biosynthesis. Mainly involved in the genes of the IAM, IPyA, IAN, and TAM pathways (Table 3 and Supplementary Figure S5). *B. pyrrocinia* JK-SH007 contains all the genes required for IAM and TAM pathways, but lacks the key genes for IPyA and IAN pathways. Explain that there is no complete IPyA or IAN pathway in *B. pyrrocinia* JK-SH007.

**TABLE 3:** Genomic analysis of indole-3-acetic acid (IAA) biosynthesis pathways in *B. pyrrocinia* JK-SH007.

**Pathpay**	**Enzyme**	***B. pyrrocinia* JK-SH007**
Indole-3-acetamide (IAM)	Trypthophan 2-monooxygenase (EC:1.13.12.3)	+
	Amidase (EC:3.5.1.4)	+
Indole-3-pyruvic acid (IPyA)	Trp aminotransferase (EC:2.6.1.27)	−
	Indolepyruvate decarboxylase (EC:4.1.1.74)	−
	Aldehyde dehydrogenase (EC:1.2.1.3)	+
Indole-3-acetonitrile (IAN)	Trp-2-monoxigenase (EC: 1.13.12.3)	+
	Nitrile hidratase (EC:4.2.1.84)	−
	Nitrilase (EC:3.5.5.1)	−
Tryptamine (TAM)	Trp decarboxylase (EC:4.1.1.28)	+
	Monoamine oxidase (EC:1.4.3.4)	+
	Aldehyde dehydrogenase (EC:1.2.1.3)	+

*+, sequence identified; −, sequence not identified.*

### Relationship Between Different Growth Periods of *B. pyrrocinia* JK-SH007 and IAA Biosynthesis

**FIGURE 1:**
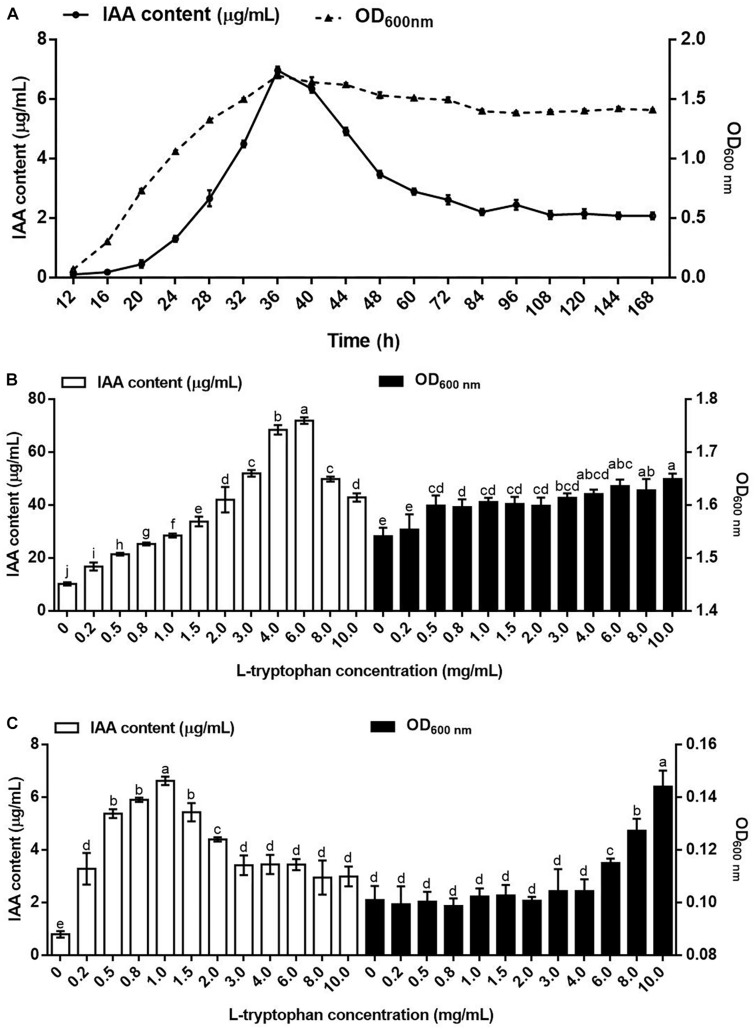
Indole-3-acetic acid in *B. pyrrocinia* JK-SH007. **(A)** Relationship between the growth of *B. pyrrocinia* JK-SH007 and IAA biosynthesis. **(B)** Effects of L-Trp on the synthesis of IAA by *B. pyrrocinia* JK-SH007 in TSB liquid medium. **(C)** Effects of L-Trp on the synthesis of IAA by *B. pyrrocinia* JK-SH007 in SD liquid medium. The error bars represent the SDs, and different letters indicate significant differences.

The relationship between the IAA yield and the growth curve of *B. pyrrocinia* JK-SH007 was assessed (Figure 1A), and the increases in the number of bacteria at the early stage gradually increased the amount of IAA synthesized by *B. pyrrocinia* JK-SH007. During the culture of *B. pyrrocinia* JK-SH007, the amount of IAA synthesized at the stationary phase reached a maximum of 6.969 μg/mL at 36h and then decreased rapidly, which may be due to the degradation of IAA by *B. pyrrocinia* JK-SH007 itself.

### Effect of L-Trp Concentration on IAA Production

Within a specific range, with increasing concentrations of L-Trp, the ability of *B. pyrrocinia* JK-SH007 in TSB liquid medium to synthesize IAA continuously improved. Although the amount of IAA synthesized by *B. pyrrocinia* JK-SH007 decreased when the L-Trp concentration reached 8.0 and 10.0 mg/mL, it was still higher than that produced without L-Trp (Figure 1B).

In the absence of L-Trp in the SD liquid medium, *B. pyrrocinia* JK-SH007 produced very small amounts of IAA (0.795 μg/mL). Increases in the L-Trp concentration within a specific range improved the ability of *B. pyrrocinia* JK-SH007 to synthesize IAA, until the maximum IAA concentration of 6.621 μg/mL was obtained with an L-Trp concentration of 1.0 mg/mL. Although further increases in the L-Trp concentrations to levels above 1.5 mg/mL decreased the amount of IAA synthesized by *B. pyrrocinia* JK-SH007, this level of IAA produced remained higher than that produced in the absence of L-Trp (Figure 1C). The difference of IAA yield of *B. pyrrocinia* JK-SH007 between TSB medium and SD medium may be due to the lack of amino acids in SD medium, which leads to the fact that the growth state of *B. pyrrocinia* JK-SH007 is much lower than that of TSB medium, so it limits the production of *B. pyrrocinia* JK-SH007 IAA. However, in both media, L-Trp promoted the growth of *B. pyrrocinia* JK-SH007 to a certain extent. Overall, the results revealed that L-Trp exerted a very significant (*P* < 0.05) effect on the synthesis of IAA by *B. pyrrocinia* JK-SH007, which indicates that L-Trp is an important precursor in the synthesis of IAA by *B. pyrrocinia* JK-SH007. The results also showed that the amount of IAA synthesized by *B. pyrrocinia* JK-SH007 was related to its growth state.

### Effects of Temperature and pH on the Synthesis of IAA by *B. pyrrocinia* JK-SH007

**FIGURE 2:**
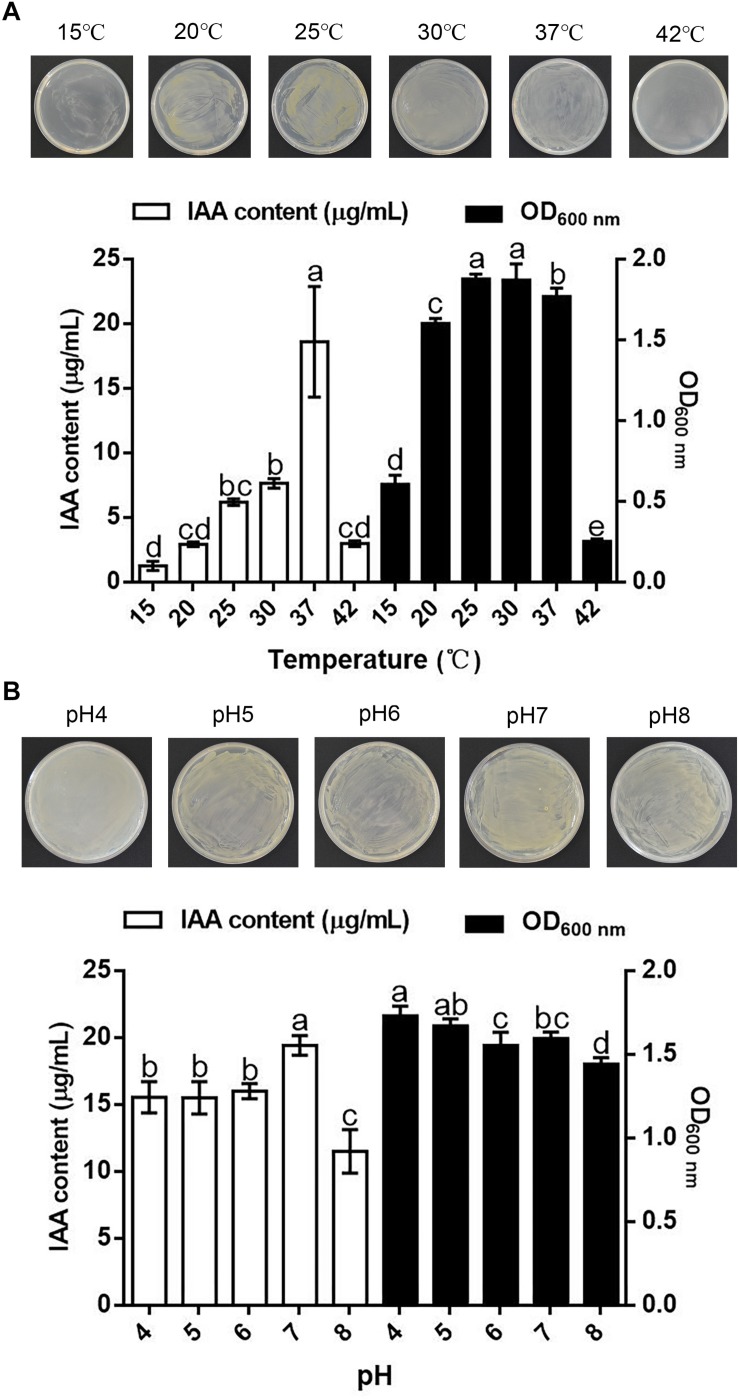
Effect of temperature and pH on the synthesis of IAA by *B. pyrrocinia* JK-SH007. **(A)** Effect of temperature on the synthesis of IAA by *B. pyrrocinia* JK-SH007. **(B)** Effect of pH on the synthesis of IAA by *B. pyrrocinia* JK-SH007. The error bars represent the SDs, and different letters indicate significant differences.

Temperature substantially influenced the synthesis of IAA by and the growth of *B. pyrrocinia* JK-SH007. The results showed that *B. pyrrocinia* JK-SH007 could grow in the temperature range of 15–42°C, but temperatures that were too high or low were not conducive to the growth of *B. pyrrocinia* JK-SH007 or to IAA production. *B. pyrrocinia* JK-SH007 grew optimally at 25 and 30°C, but a temperature of 37°C resulted in an IAA yield of 18.608 μg/mL, which was significantly (*P* < 0.05) higher than that reached with the other temperatures (Figure 2A). The growth of *B. pyrrocinia* JK-SH007 on TSB agar plates was the same as that on TSB liquid medium.

The results showed that pH exreted a certain effect on the synthesis of IAA by and the growth of *B. pyrrocinia* JK-SH007. The strain was able to grow over a broad pH range of 4–8, and the growth of *B. pyrrocinia* JK-SH007 on TSB agar plates was the same as that on TSB liquid medium. The highest IAA production yield was 19.432 μg/mL was reached with a pH value of 7, whereas a 40.77% lower yieldwas obtained at pH 8, and the fastest growth of *B. pyrrocinia* JK-SH007 was observed at pH 4 (Figure 2B).

### Prokaryotic Expression of Tryptophan 2-Monooxygenase and Indoleacetamide Hydrolase

#### Construction of the Prokaryotic Expression Vector

The *iaaM* (GenBank accession MK238350) and *iaaH* genes (GenBank accession MK238351) were obtained by using *B. pyrrocinia* JK-SH007 genomic DNA as the template for the design of a primer with a restriction enzyme site (Supplementary Figures S6A,B). The PCR product obtained after restriction enzyme digestion was ligated with the digested expression vector pET32a and transformed into *E. coli* BL21 (DE3). After colony PCR verification (Supplementary Figures S6C,D), the recombinant plasmid was extracted and verified by double-enzyme digestion (Supplementary Figures S6E,F). The positive recombinant plasmid was also verified by sequencing. The correct pET32a-*iaaM/iaaH* recombinant plasmid (Supplementary Figures S6G,H) was therefore used in the subsequent experiments.

#### Induced Expression and SDS-PAGE Analysis of Recombinant Proteins

**FIGURE 3:**
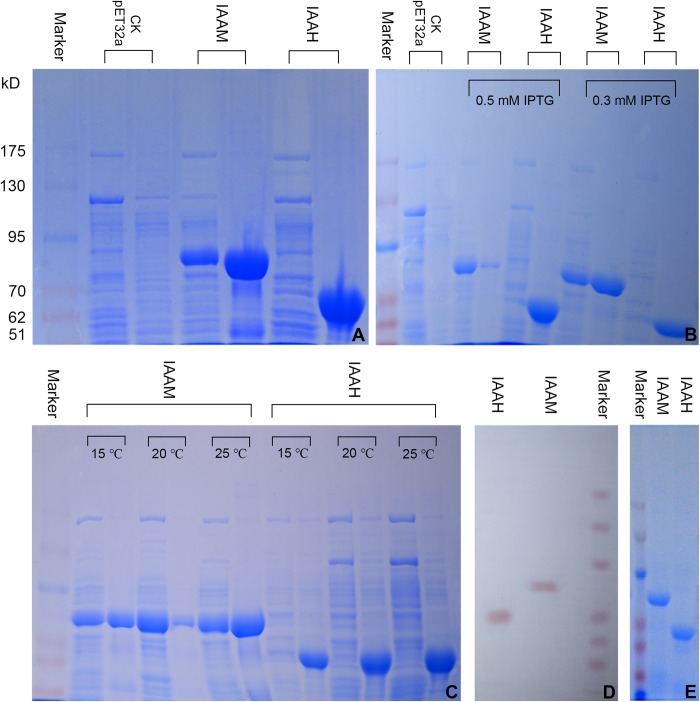
Prokaryotic expression, purification and Western blot identification of the tryptophan 2-monooxygenase (IAAM) and indoleacetamide hydrolase (IAAH) fusion proteins. **(A)** Expression of tryptophan 2-monooxygenase and indoleacetamide hydrolase (Opti-Protein Marker, the sizes from top to bottom are as follows: 175, 130, 95, 70, 62, and 51 kD; for each treatment, the left lane is the supernatant and the right is the precipitate; this information is also relevant to later figures). **(B)** Effect of the IPTG concentration on the expression of tryptophan 2-monooxygenase and indoleacetamide hydrolase. **(C)** Effect of the temperature on the expression of the tryptophan 2-monooxygenase and indoleacetamide hydrolase fusion proteins. **(D)** Western blot analysis of tryptophan 2-monooxygenase and indoleacetamide hydrolase. **(E)** Purified tryptophan 2-monooxygenase and indoleacetamide hydrolase.

The expression of the tryptophan 2-monooxygenase and indoleacetamide hydrolase fusion proteins was analyzed by 6% SDS-PAGE. As shown in Figure 3A, bands consistent with the tryptophan 2-monooxygenase fusion protein (approximately 80 kD) can be observed in the supernatant and precipitate, whereas bands consistent with the indoleacetamide hydrolase fusion protein (approximately 60 kD) can be observed in the precipitate. The results indicated that the tryptophan 2-monooxygenase and indoleacetamide hydrolase fusion proteins were both expressed in *E. coli* BL21 (DE3), and the tryptophan 2-monooxygenase fusion protein was expressed in the supernatant and precipitate, mostly in the form of inclusion bodies, whereas the indoleacetamide hydrolase fusion protein was expressed mostly in inclusion bodies, but hardly in the supernatant (Figure 3A).

The expression of the tryptophan 2-monooxygenase and indoleacetamide hydrolase fusion proteins was induced by IPTG at concentrations of 0.3 and 0.5 mM (Figure 3B). The expression of the tryptophan 2-monooxygenase fusion protein at an IPTG concentration of 0.5 mM was significantly higher than that obtained with an IPTG concentration of 0.3 mM. Although the expression of the indoleacetamide hydrolase fusion protein pbtained with an IPTG concentration of 0.5 mM was higher than that at an IPTG concentration of 0.3 mM, it was expressed solely in inclusion bodies.

The induced expression of the tryptophan 2-monooxygenase and indoleacetamide hydrolase fusion proteins at temperatures of 15, 20, and 25°C is shown in Figure 3C. The expression level of the tryptophan 2-monooxygenase fusion protein in the supernatant obtained with a temperature of 20°C than at 15 and 25°C. However, the indoleacetamide hydrolase fusion protein was expressed in large quantities at 15, 20, and 25°C but was present only in inclusion bodies. Therefore, expression of the tryptophan 2-monooxygenase and indoleacetamide hydrolase fusion proteins was induced by IPTG at a concentration of 0.5 mM and a temperature of 20°C to facilitate purification and subsequent experiments.

#### Western Blot Analysis

A Western blot analysis with His monoclonal antibody showed that the tryptophan 2-monooxygenase and indoleacetamide hydrolase (Figure 3D) fusion proteins induced by IPTG formed distinct protein imprinting bands at 80 kD and 60 kD, respectively, which confirmed the expression of the fusion proteins.

#### Purification and Renaturation of Tryptophan 2-Monooxygenase and Indoleacetamide Hydrolase

An IPTG concentration of 0.5 mM and a temperature of 20°C resulted in high expression of the tryptophan 2-monooxygenase and indoleacetamide hydrolase fusion proteins. The supernatant of the tryptophan 2-monooxygenase fusion protein and the inclusion bodies of the indoleacetamide hydrolase fusion protein were collected, and the indoleacetamide hydrolase fusion protein inclusion bodies were dissolved in a buffer containing 8 M urea. The tryptophan 2-monooxygenase and indoleacetamide hydrolase fusion proteins were purified on a Ni-IDA-Sefinose column. The purified proteins were analyzed by 6% SDS-PAGE. As shown in Figure 3E, single bands appeared in the two lanes, which indicated the successful purification of the tryptophan 2-monooxygenase and indoleacetamide hydrolase (Figure 3E) fusion proteins. The purified indoleacetamide hydrolase fusion protein inclusion bodies were renatured by dialysis refolding. The tryptophan 2-monooxygenase and indoleacetamide hydrolase fusion proteins were concentrated by ultrafiltration, and the concentration of each protein was determined using the BCA method. The concentration of the tryptophan 2-monooxygenase fusion protein was 635.616 μg/mL, and the concentration of the indoleacetamide hydrolase fusion protein was 772.325 μg/mL.

#### Characterization of the Tryptophan 2-Monooxygenase and Indoleacetamide Hydrolase Enzymes

HPLC-MS/MS analysis (Table 4) showed that 2.015 ng/mL IAM (Supplementary Figure S7A) was generated in the product mixture obtained from the tryptophan 2-monooxygenase and L-Trp, and 25.645 ng/mL IAA (Supplementary Figure S7B) was generated from the reaction of indoleacetamide hydrolase and IAM. These findings indicate that the prokaryotic-expressed purified tryptophan 2-monooxygenase and indoleacetamide hydrolase enzymes were active and that the IAM-mediated IAA synthesis pathway plays a role in *B. pyrrocinia* JK-SH007.

**TABLE 4:** HPLC-MS/MS analysis of indole-3-acetamide (IAM) and indole-3-acetic acid (IAA) in protein mixture.

**Indolic compounds**		**Indole-3-acetamide (IAM)**	**Indole-3-acetic acid (IAA)**
Mass-to-charge ratio (M/Z)		175.0 ≥130.0	176.0 ≥130.0
Acqusion	Standard	4.059	5.187
time (min)	Sample	4.134	5.198
Concentration (ng/mL)^a^		2.015 ± 0.641	25.645 ± 1.660

*^*a*^Values are average ± *SD* (*n* = 3).*

## Discussion

The HPLC-MS/MS evaluation of the *B. pyrrocinia* JK-SH007 fermentation broth revealed that *B. pyrrocinia* JK-SH007 produces IAA, IAM, IPyA, IAN, and TAM, and in the experiment assessing the production of IAA by the *B. pyrrocinia* JK-SH007 cell-free extract, the total enzyme content, including the IAA biosynthetic enzymes, was obtained by breaking the bacterial cells through ultrasonic treatment, and the obtained cell-free extract reacted with the IAA synthesis precursor L-Trp to produce IAM, IPyA, IAN, TAM, and IAA (Shokri and Emtiazi, 2010), which further indicated that the tryptophan-dependent pathway plays an important role in the synthesis of IAA by *B. pyrrocinia* JK-SH007. However, the whole genome analysis and annotation of *B. pyrrocinia* JK-SH007 showed that it did not have the key genes of IPyA and IAN pathways, and contained the integrity of IAM and TAM pathways, indicating that *B. pyrrocinia* JK-SH007 might synthesize IAA through complete IAM and TAM pathways, rather than IPyA and IAN pathways.

The relationship between IAA synthesis by and the growth of *B. pyrrocinia* JK-SH007 was determined. The results showed that the amount of IAA synthesized by *B. pyrrocinia* JK-SH007 increased with increases in the number of *B. pyrrocinia* JK-SH007 bacteria at the early growth stage, potentially because IAA is a secondary metabolite. The highest amount of synthesized IAA was achieved once *B. pyrrocinia* JK-SH007 reached the stationary stage, which was also consistent with the results reported by Blinkov et al. (2014). The amount of IAA produced by *B. pyrrocinia* JK-SH007 decreased gradually after 36 h of cultivation, which might be due to the degradation of IAA by *B. pyrrocinia* JK-SH007 itself. Some studies have showed that some bacterial strains such as *Paraburkholderia phytofirmans*, *Burkholderia phytofirmans* use IAA as a sole carbon and energy source, and the degradation of IAA is important in plant growth promotion (Zúñiga et al., 2013; Donoso et al., 2017). A small amount of IAA could be synthesized by *B. pyrrocinia* JK-SH007 without the addition of exogenous L-Trp, and L-Trp has been detected as the main compound in some plant secretions (Kamilova et al., 2006). Within a specific concentration range, L-Trp significantly enhanced the ability of *B. pyrrocinia* JK-SH007 to synthesize IAA, and L-Trp is an important physiological precursor for the synthesis of IAA by *B. pyrrocinia* JK-SH007. This finding also proves that some microbes can produce a small amount of IAA in the absence of L-Trp but can generate a relatively large amount of IAA in the presence of IAA (Khalid et al., 2004; Zahir et al., 2010).

The optimal temperature and pH for IAA synthesis by *B. pyrrocinia* JK-SH007 were 37°C and 7, respectively. The activities of enzymes associated with the IAA biosynthesis pathway in *B. pyrrocinia* JK-SH007 were higher under these conditions than under the other tested conditions, which enhanced the production of IAA; however, the optimal growth conditions for *B. pyrrocinia* JK-SH007 were 25°C or 30°C and a pH of 4, which indicated that there was no direct relationship between the biosynthesis of IAA by *B. pyrrocinia* JK-SH007 and the optimal growth of its cells. The optimal temperature and pH for IAA production can reflect the ecological sites occupied by bacteria, at various temperatures and under the slightly acidic conditions between roots and the physiological conditions within plants (Duca et al., 2014). The above described experiments also showed that the yield of IAA was affected by the culture conditions, growth conditions, and substrate availability (Chaiharn and Lumyong, 2010).

Through the prokaryotic expression and characterization of tryptophan 2-monooxygenase and indoleacetamide hydrolase in *B. pyrrocinia* JK-SH007, this study proved that the IAM pathway is active in *B. pyrrocinia* JK-SH007, and the detection of IAM in the fermentation broth of *B. pyrrocinia* JK-SH007 confirmed this conclusion. However, other IAA synthesis intermediates, namely, IPyA, IAN and TAM were also detected in the fermentation broth of *B. pyrrocinia* JK-SH007. And the whole genome of *B. pyrrocinia* JK-SH007 does not have the key genes of IPyA and IAN pathways, but contains the integrity of IAM and TAM pathways, *B. pyrrocinia* JK-SH007 might also synthesize IAA through the TAM pathway, but further functional verification of these IAA synthesis pathways is needed. In fact, PGPB contain multiple IAA pathways (Valérie et al., 2007).

We demonstrated that the PGPB *B. pyrrocinia* JK-SH007 can produce the plant auxin IAA through the IAM pathway. This discovery not only extends the understanding of the IAM-mediated IAA synthesis pathway in *Burkholderia cepacia* but also indicates that the IAM-mediated IAA biosynthesis pathway has no clear boundary between plant pathogenic bacteria and PGPB (Patten and Glick, 2002a, b; Lin and Xu, 2013). The IAM-mediated IAA synthesis pathways have been observed in symbiotic bacteria such as *Azospirillum* spp., *Rhizobium* spp., and *Bradyrhizobium* spp. (Theunis et al., 2004; Malhotra and Srivastava, 2006; Spaepen and Vanderleyden, 2011).

## Conclusion

In this study, the ability of *B. pyrrocinia* JK-SH007 to synthesize the auxin IAA was confirmed by HPLC-MS/MS, and the related IAA synthetic intermediates were also detected, IAA, and its synthetic intermediates were also found in the cell-free extraction of *B. pyrrocinia* JK-SH007, but the genomic analysis of *B. pyrrocinia* JK-SH007 indicated that IAA biosynthesis was mainly through the IAM and TAM pathways. The effects of L-Trp, temperature and pH on the production of IAA by *B. pyrrocinia* JK-SH007 were studied, and the results indicate that these factors might play a role in the interaction between plants and bacteria. Through the protein expression of tryptophan 2-monooxygenase and indoleacetamide hydrolase and the verification of their activities, this study proved that the IAM pathway exists in *B. pyrrocinia* JK-SH007 and plays an important role in the synthesis of IAA. These findings verify that an IAA biosynthesis pathway based on IAM is found in *B. pyrrocinia* JK-SH007 and that L-Trp is the precursor of this pathway. Future studies should further investigate the overall IAA biosynthesis pathway in *B. pyrrocinia* JK-SH007 and the interaction between IAA biosynthesis pathways. Obtaining an in-depth understanding of the growth-promoting mechanism of *B. pyrrocinia* JK-SH007 is necessary for the application of its growth-promoting function. This study also provides a reference for related research on endophytic bacteria that can synthesize the auxin IAA in plants to promote plant growth without causing host diseases.

## Data Availability Statement

The datasets generated for this study can be found in the GenBank accession MK238350 and GenBank accession MK238351.

## Author Contributions

W-HL, F-FC, J-RY, and C-EW performed the research. W-HL, H-HF, X-QF, and J-YS analyzed the data. All authors wrote the manuscript.

## Conflict of Interest

The authors declare that the research was conducted in the absence of any commercial or financial relationships that could be construed as a potential conflict of interest.
