# Supplementary material for: Indole-3-Acetic Acid in Burkholderia pyrrocinia JK-SH007: Enzymatic Identification of the Indole-3-Acetamide Synthesis Pathway
Source: Front Microbiol. 2019 Nov 5;10:2559. doi: 10.3389/fmicb.2019.02559 (PMC6848275; doi:10.3389/fmicb.2019.02559)
Supplement: Supplementary file 1 [file Data_Sheet_1.pdf]

Indole-3-Acetic Acid in *Burkholderia pyrrocinia* JK-SH007: Enzymatic Identification of the Indole-3-Acetamide Synthesis Pathway

Wan-Hui Liu, Fei-Fei Chen, Chao-En Wang, Huan-Huan Fu, Xue-Qi Fang, Jian-Ren Ye* and Ji-Yuan Shi

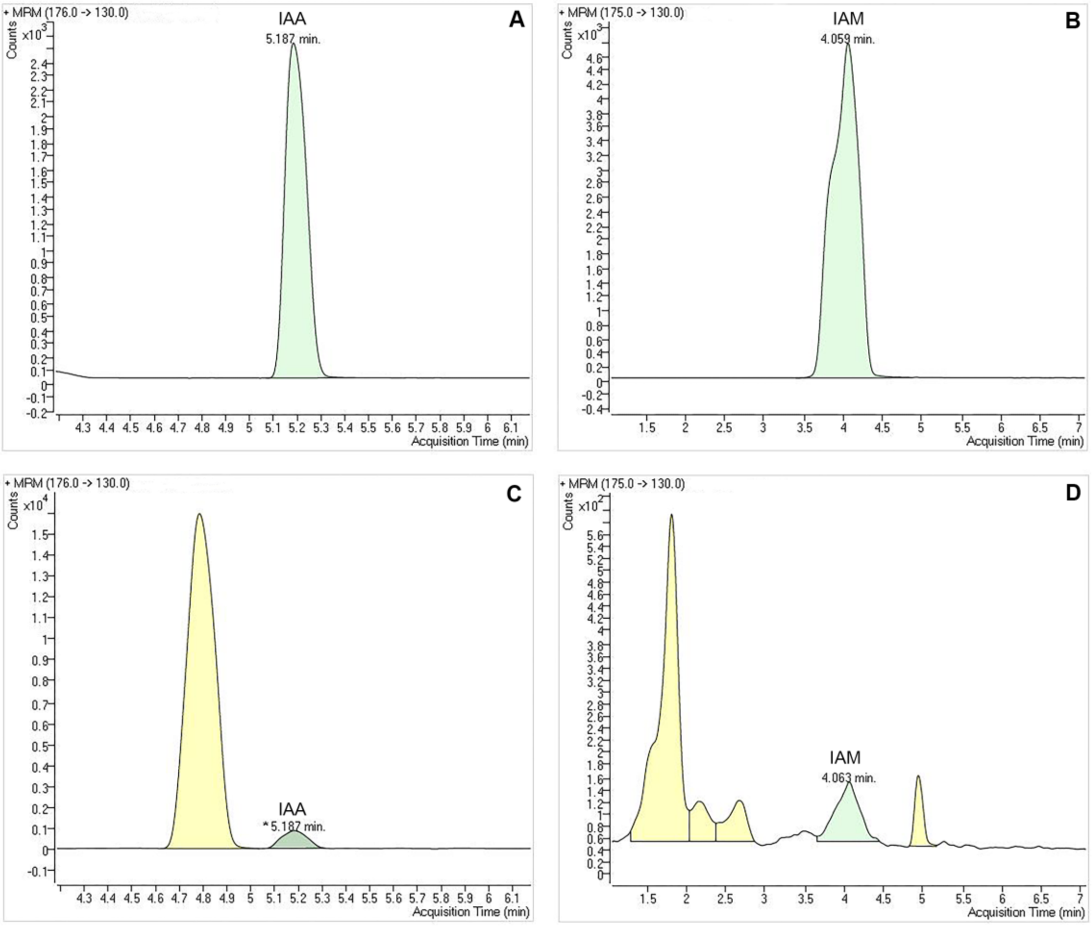

FIGURE. S1. Mass spectra of IAA and IAM produced by *B. pyrrocinia* JK-SH007. The spectra were obtained by HPLC-MS/MS. **(A)** Mass spectrum of the standard sample of IAA. **(B)** Mass spectrum of the standard sample of IAM. **(C)** Mass spectrum of IAA produced by *B. pyrrocinia* JK-SH007**. (D)** Mass spectrum of IAM produced by *B. pyrrocinia* JK-SH007.

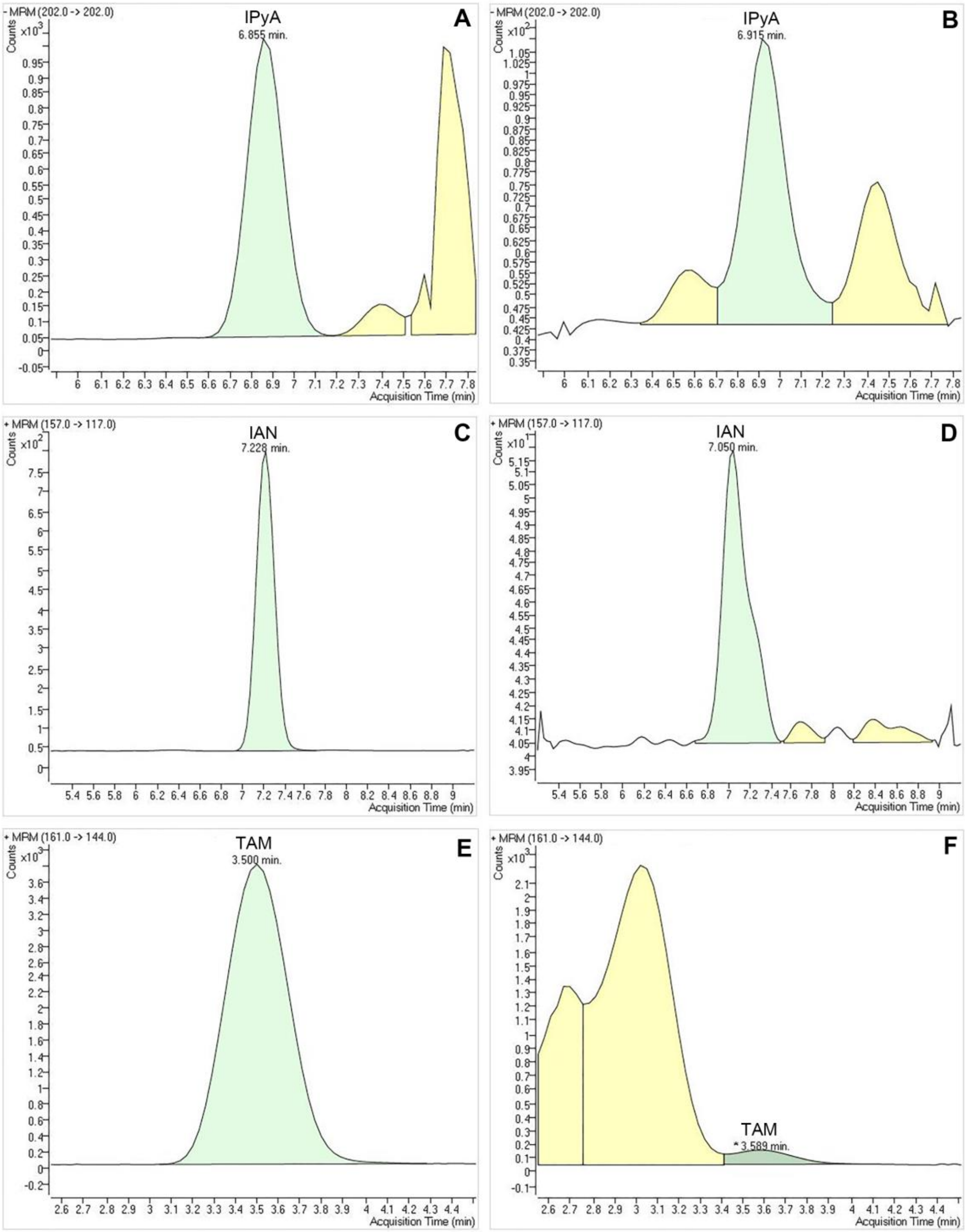

FIGURE. S2. Mass spectrum of IAA synthesis intermediates produced by *B. pyrrocinia* JK-SH007. The spectra were obtained by HPLC-MS/MS. **(A)** Mass spectrum of the standard sample of IPyA. (**B)** Mass spectrum of IPyA produced by *B. pyrrocinia* JK-SH007**. (C)** Mass spectrum of the standard sample of IAN. **(D)** Mass spectrum of IAN produced by *B. pyrrocinia* JK-SH007. **(E)** Mass spectrum of the standard sample of TAM. **(F)** Mass spectrum of TAM produced by *B. pyrrocinia* JK-SH007.

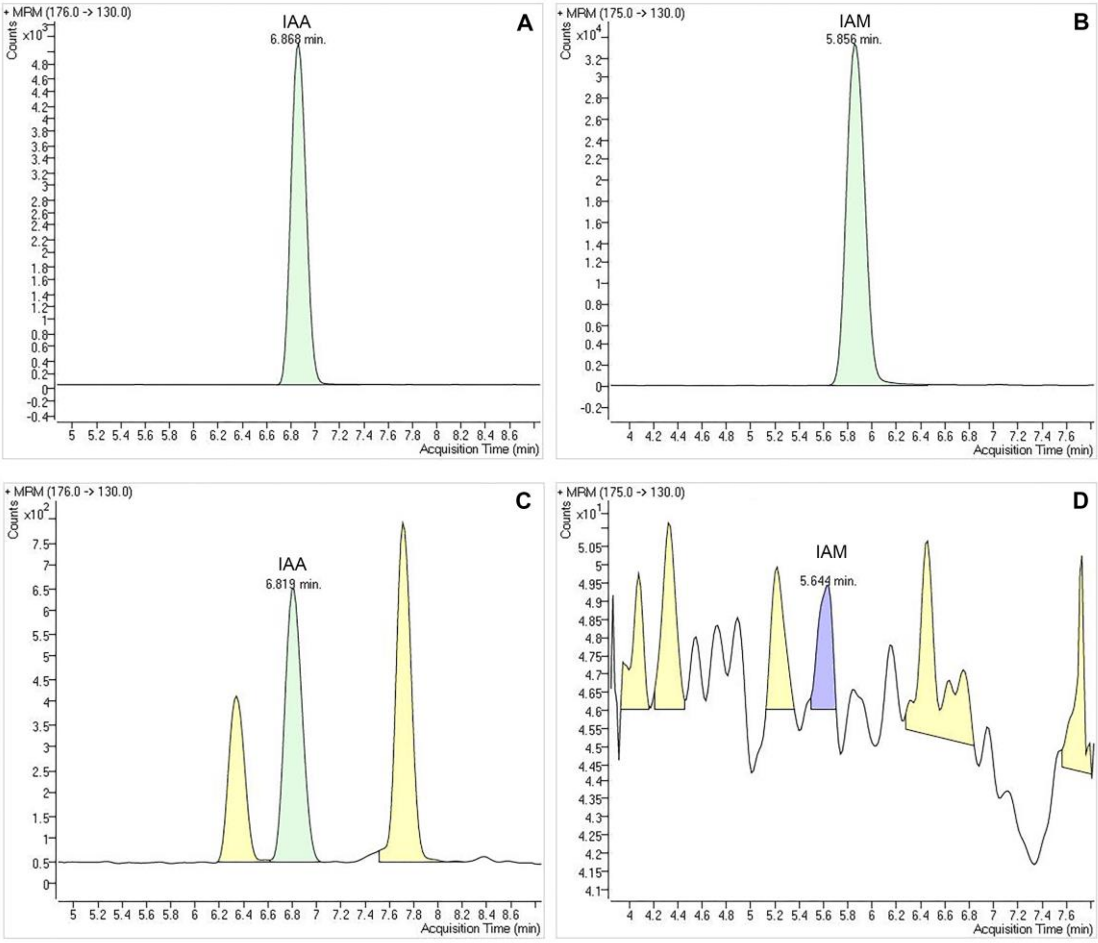

FIGURE. S3. IAA and IAM production in the cell-free extract of *B. pyrrocinia* JK-SH007. **(A)** Mass spectrum of the standard sample of IAA. **(B)** Mass spectrum of the standard sample of IAM. **(C)** Mass spectrum of IAA produced by the cell-free extract of *B. pyrrocinia* JK-SH007**. (D)** Mass spectrum of IAM produced by the cell-free extract of *B. pyrrocinia* JK-SH007.

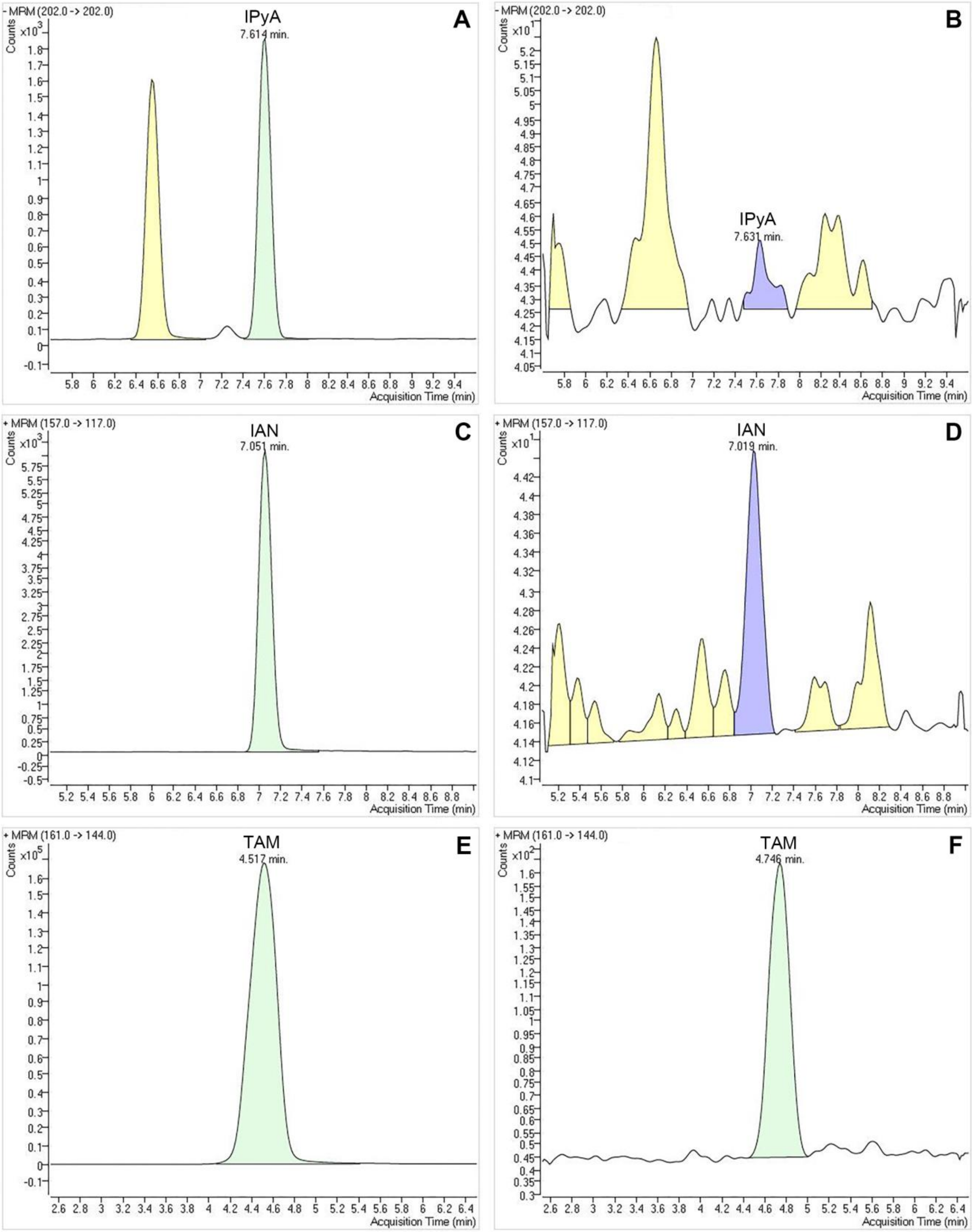

FIGURE. S4. Production of IAA synthesis intermediates in cell-free extract of *B. pyrrocinia* JK-SH007. **(A)** Mass spectrum of the standard sample of IPyA. **(B)** Mass spectrum of IPyA. **(C)** Mass spectrum of the standard sample of IAN. **(D)** Mass spectrum of IAN**. (E)** Mass spectrum of the standard sample of TAM. **(F)** Mass spectrum of TAM.

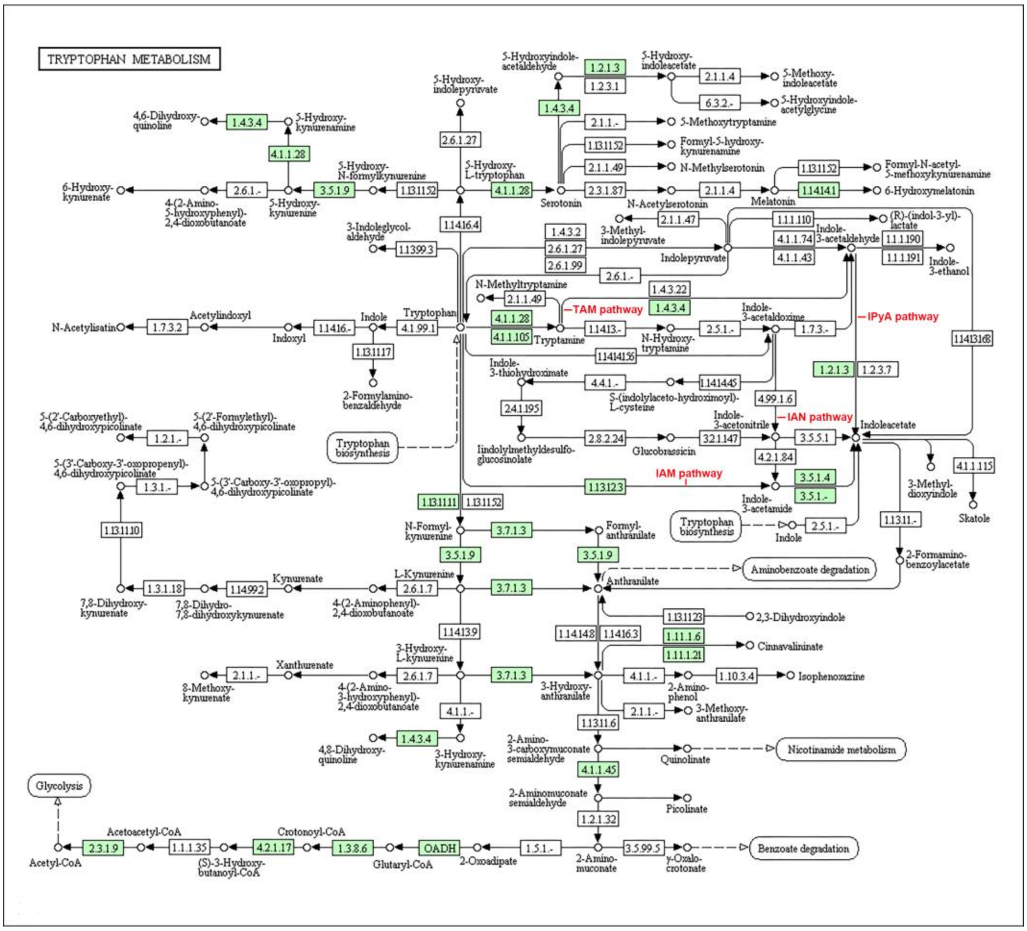

FIGURE. S5. Putative "KEGG pathway" of trp metabolism and IAA biosynthetic pathways in the *B. pyrrocinia* JK-SH007. Green-labeled putative genes associated with the IAA synthesis pathways recognized in the *B. pyrrocinia* JK-SH007 genome.

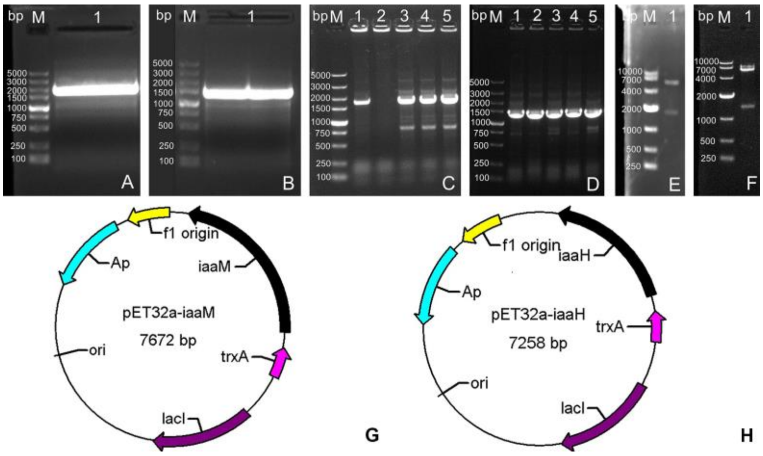

FIGURE. S6. **(A)** Amplification of the *iaaM* gene by PCR. **(B)** Amplification of the *iaaH* gene by PCR. **(C)** PCR identification of *iaaM*-positive clones. **(D)** PCR identification of *iaaH*-positive clones. **(E)** Double-digestion validation of the recombinant plasmid pET32a-*iaaM*. **(F)** Double-digestion validation of the recombinant plasmid pET32a-*iaaH*. **(G)** Maps of the recombinant plasmid pET32a-*iaaM*. **(H)** Maps of the recombinant plasmid pET32a-*iaaH* (drawn using Winplas 2.7).

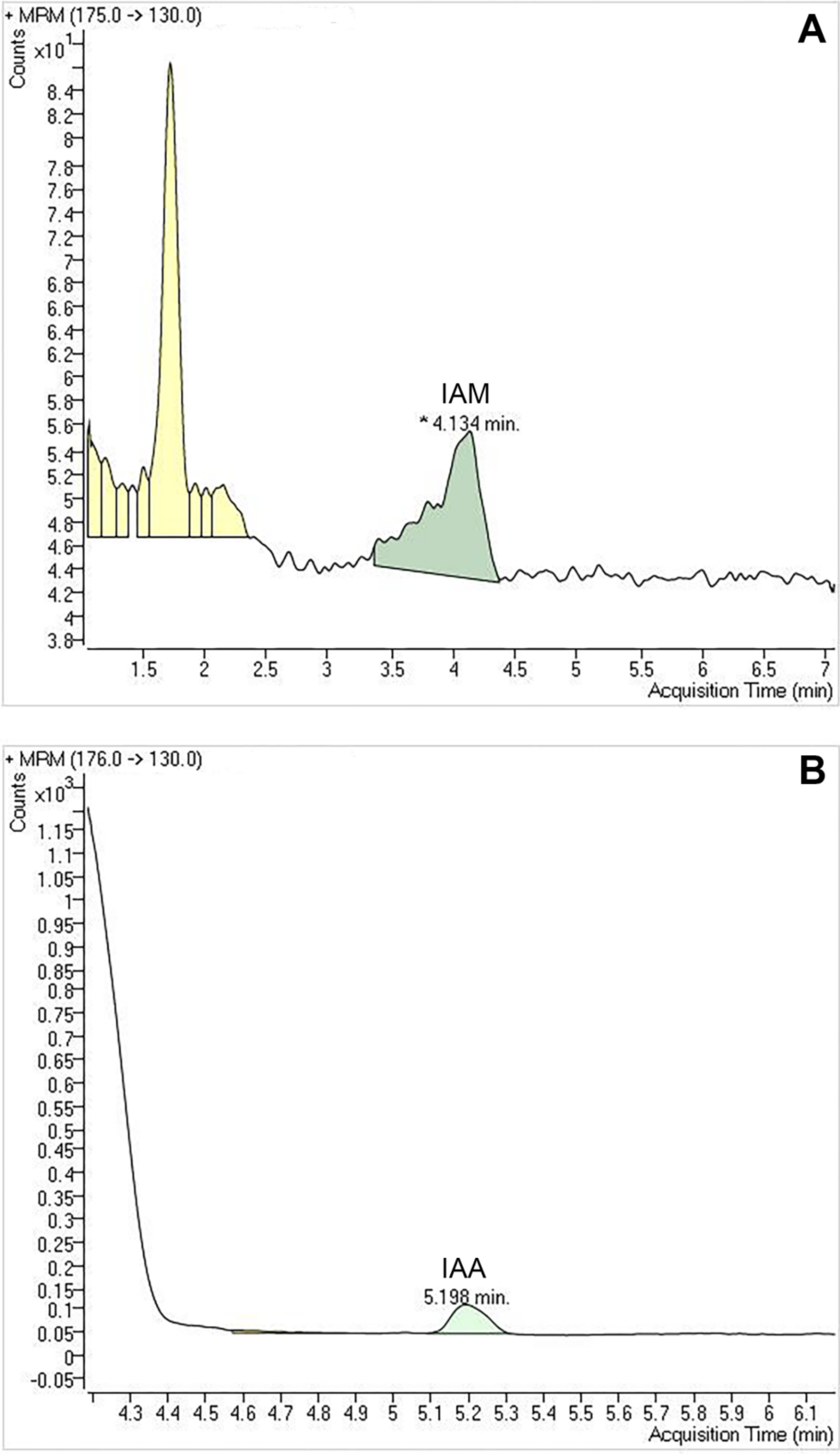

FIGURE. S7. IAA and IAM determination through HPLC-MS/MS. **(A)** Mass spectrum of IAM (standard reference figure S1B). **(B)** Mass spectrum of IAA (standard reference figure S1A).
